# National Seroprevalence of *Coxiella burnetii* in Chile, 2016–2017

**DOI:** 10.3390/pathogens10050531

**Published:** 2021-04-28

**Authors:** Teresa Tapia, María Fernanda Olivares, John Stenos, Rodrigo Iglesias, Nora Díaz, Natalia Vergara, Viviana Sotomayor, Doris Gallegos, Ricardo J Soares Magalhães, Johanna Acevedo, Pamela Araya, Stephen R Graves, Juan Carlos Hormazabal

**Affiliations:** 1 Bacteriology, Sub-department of Infectious Diseases, Department Biomedical Laboratory, Public Health Institute of Chile, Santiago 7780050, Chile; ttapia@ispch.cl (T.T.); riglesias@ispch.cl (R.I.); paraya@ispch.cl (P.A.); 2 Departamento de Epidemiología, DIPLAS, Ministerio de Salud de Chile, Santiago 8320123, Chile; maria.olivares@minsal.cl (M.F.O.); noraluz07@gmail.com (N.D.); nvergara@minsal.cl (N.V.); vsotomay@minsal.cl (V.S.); dgallego@minsal.cl (D.G.); johanna.acevedo@minsal.cl (J.A.); 3 Australian Rickettsial Reference Laboratory, Geelong University Hospital, Geelong, VIC 3220, Australia; JOHN.STENOS@barwonhealth.org.au (J.S.); graves.rickettsia@gmail.com (S.R.G.); 4 Children’s Health and Environment Program, Child Health Research Centre, The University of Queensland, South Brisbane, QLD 4101, Australia; r.magalhaes@uq.edu.au; 5 Spatial Epidemiology Laboratory, School of Veterinary Science, The University of Queensland, Gatton, QLD 4343, Australia

**Keywords:** Chile, *Coxiella burnetii*, Q fever, seroprevalence, zoonotic disease

## Abstract

*Coxiella burnetii* is an intracellular bacterium and the cause of the zoonotic infection, Q fever. National surveillance data on *C. burnetii* seroprevalence is currently not available for any South American country, making efforts of public health to implement strategies to mitigate infections in different at-risk groups within the population extremely challenging. In the current study, we used two commercial anti-*C. burnetii* immunoassays to screen sera collected from a sample of the Chilean population as part of a 2016–2017 national health survey (*n* = 5166), nationwide and age-standardized. The seroprevalence for *C. burnetii* for persons ≥ 15 years was estimated to be 3.0% (95% CI 2.2–4.0), a level similar to national surveys from The Netherlands (2.4%) and USA (3.1%), but lower than Australia (5.6%). A linear increase of *C. burnetii* seropositivity was associated with an individual’s age, with the peak seroprevalence 5.6% (95% CI 3.6–8.6) observed in the ≥65 years’ group. *C. burnetii* seropositivity was significantly higher in the southern macro-zone 6.0% (95% CI 3.3–10.6) compared to metropolitan region 1.8% (95% CI 0.9–3.3), the former region being home to significant livestock industries, particularly dairy farming. These data will be useful to inform targeted strategies for the prevention of Q fever in at-risk populations in Chile.

## 1. Introduction

Q fever is a systemic human disease resulting from infection by the intracellular Gram-negative and pleomorphic coccobacillus, *Coxiella burnetii* [1,2]. While most infections are asymptomatic, clinically, Q fever can be classified as either an acute or chronic disease. Symptoms of acute illnesses include fever, chills, headache, fatigue, malaise and myalgia, an unspecific range of symptoms that can often be confused with other respiratory-like illnesses, requiring completely different treatment strategies. In severe cases, an acute infection may progress to atypical aggressive pneumonia or hepatitis [1,3]. Although rare (2–5%), chronic infection has been associated with endocarditis or vascular damage. The development of chronic fatigue syndrome may occur after acute or chronic Q fever [1,4].

The primary reservoirs of *C. burnetii* include ruminants (goats, sheep, and cattle) and other domesticated and companion mammals, wild mammals, marine mammals and ticks [5,6]. The broad host range of *C. burnetii* is an important factor for the environmental risk of exposure [5]. Transmission to humans normally occurs by inhalation of contaminated aerosols or through direct contact with infectious animal birth materials [7,8]. The main populations at risk of Q fever are people working in the livestock production sector (e.g., veterinarians and livestock handlers), abattoir workers and people residing in rural areas or in close contact with farmed and wild animals [9,10].

Because most *C. burnetii* infections are asymptomatic, national seroprevalence surveys provide a way of measuring past exposure by detecting the presence of antibodies against *C. burnetii*. This pathogen exists as one of two forms of antigens, called phases I and phases II, generating different patterns of the antibody response infection [1]. Antibodies (IgM and/or IgG) against antigens in phase II of *C. burnetii* are expressed early during acute illness. On the other hand, high-titer anti-phase I antibodies are associated with chronic Q fever infection [1]. To date, there have been a small number of nationally representative seroprevalence surveys across all age groups. In the Netherlands, home to the largest outbreak of documented Q fever among a human population between 2007 and 2010 [11], a seroprevalence of 2.4% (*n* = 5654) was reported pre-outbreak [12]. Similar seroprevalence levels have been reported in Australia 5.6% (*n* = 2785) [13], USA 3.1% (*n* = 4437) [14] and Bhutan 6.9% (*n* = 864) [15]. An exception to these studies are Northern Ireland, with 12.8% (*n* = 2394) [16] and Cyprus a 52.7% (*n* = 583) [17]. In South America, few studies on human Q fever have been reported [18]. Outbreaks of febrile illness have been reported in Colombia, Uruguay, Argentina and Peru [18]. Seroprevalence studies were reported in groups of individuals with a high risk associated with their occupational risk group in Ecuador (seroprevalence of 34% in farm workers) [19] and in cases of community-acquired pneumonia in French Guiana, (24.4% seroprevalence) [20]. Traditionally, surveillance of Q fever in Latin American countries has been challenging due to the nonspecific nature and/or potential asymptomatic presentation of this disease and limitations in the availability of specific assays for testing in hospitals in the region. As a result, the incidence of Q fever in this geographic region is likely to be significantly underreported.

In Chile, Q fever became a nationally notifiable disease in 2004. However, historically, there are very few reported cases in the country. The first Q fever outbreak in Chile was reported in 1998 associated with imported lambs (unpublished data). The first small human cross-sectional serosurvey for *C. burnetii* among healthy young adults in four different regions of Chile found a seroprevalence of around 0.1% [21]. More recently, in July 2017, an outbreak of atypical pneumonia erupted in the southern regions of Chile [22]. An investigation into the sera from human cases associated with the July 2017 outbreak of atypical pneumonia conducted in three regions of southern Chile found 20% seropositivity for *C. burnetii,* providing the most compelling evidence yet of its burden in Chile. The absence of other documented cases highlights the potential underreporting of *C. burnetii* infection in Chile and the challenges associated with clinical and laboratory detection of this pathogen [22]. As a result, the Chilean Ministry of Health and the Public Health Institute of Chile recognized the need for a better understanding of the prevalence of *C. burnetii* in Chile and organized a seroprevalence survey in the general Chilean population.

The aim of this study was to determine the seroprevalence of *C. burnetii* in a representative sample of the Chilean population before the July 2017 outbreak [22] using sera arising from the 2016–2017 cycle of the nationally representative National Health Survey (Encuesta Nacional de Salud; ENS) [23].

## 2. Results

### 2.1. Serological Analysis of IgG Antibodies against Coxiella burnetii

The results of serological analysis of the Chilean population, separated by geographic region, are presented in Table 1.

Measurement of *C. burnetii* IgG phase II antibodies revealed seropositivity of 2.8% (143/5166). Fifty-nine (1.1%) were interpreted as equivocal. Of these, 30 samples were confirmed positive by IFA, and the rest of the equivocal samples were negative. One hundred and sixty (160) of the 202 positive/equivocal samples (79.2%) were then confirmed seropositive with titers ≥ 1/32 by IFA, representing an overall IgG phase I and II positivity to *C. burnetii* of 160/5166 (3.1%) in the overall sample set.

In terms of the agreement between assays, 43/160 (26.8%) had both IgG phase I and phase II antibodies detected. None of the serum samples was positive for the only phase I IgG. Discrepant results were found in 42 samples (42/202; 20.8%) tested by both ELISA and IFA and with titers < 1/32. The majority of these samples (29/42; 69.0%) were defined as “equivocal” after initial ELISA screening (Table 1).

### 2.2. Seroprevalence of Coxiella burnetii in the Chilean Population

Based on the anti-IgG *C. burnetii* screening results, the overall nationwide seroprevalence for persons ≥15 years of age was estimated to be 3.0% (95% CI 2.2–4.0). Table 2 displays the seroprevalence adjusted for sex, age range, level of education (low, middle, high), population density (urban/rural) and macro-zones (north, central, metropolitan and south). *C. burnetii* seroprevalence was higher for men 3.7% (95% CI 2.5–5.4) than women 2.2% (95% CI 1.4–3.5). *C. burnetii* seroprevalence increased with age in the Chilean population with the highest prevalence 5.6% (95% CI 3.6–8.6) recorded in persons ≥65 years of age. In terms of education level, the *C. burnetii* seroprevalence for individuals with <8 years of formal education was more than double 6.8% (95% CI 3.9–11.4) the national average. When the latter two characteristics were considered together, the relationship between individuals aged ≥65 years and with <8 years of formal education compared to other categories was statistically significant (*p* < 0.001).

We also examined the relationship between *C. burnetii* seroprevalence in the Chilean population and the population density and geographic origin of the individuals sampled. Our results indicated that *C. burnetii* seroprevalence was significantly higher in individuals from rural areas with a lower population density than those from urban regions (*p* < 0.001). *C. burnetii* seroprevalence was also higher towards the southern macro-zone with a 6.0% seroprevalence documented in this subpopulation compared to the northern and metropolitan macro-zones. Statistical analyses revealed a significant association between a rural area of origin and the central and southern Chilean macro-zones in *C. burnetii* seropositive individuals (*p* < 0.001).

**Figure 1:**
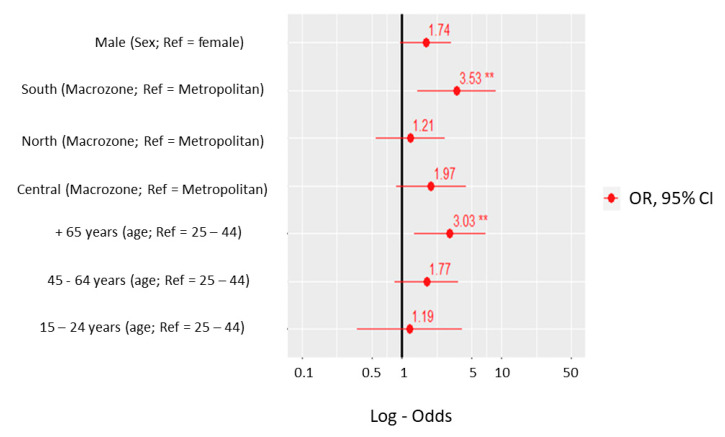
Odds ratio of *Coxiella burnetii* seropositivity adjusted for sex, age group and macro-zone according to the ENS2016-2017 survey. ** Statistically significant.

A binomial logistic regression model was developed to quantify using odds ratios (OR) the association between *C. burnetii* seropositivity and individual-level and contextual risk factors (Figure 1). Two risk factors were identified as showing significant differences. The odds of exposure in the southern macro-zone was found to be 3.5 times higher than that of the metropolitan macro-zone (OR = 3.53; 95% CI 1.4–8.7); *p* < 0.015), and the odds of exposure to *C. burnetii* was 3.03 (95% CI 1.3–6.9) times higher in individuals ≥ 65 years’ age compared to the 24–44 year age group.

## 3. Discussion

A dearth of information exists on the seroprevalence of antibodies against *C. burnetii* in countries in Latin America. Knowledge of the *C. burnetii* seroprevalence and associated risk factors will support public health stakeholders at the national level in leveraging the need for preventative actions and occupational health recommendations in consultation with the animal health sector while at the same time strengthening the clinical and laboratory capacity to diagnose this important zoonosis.

The findings from this first large nationwide survey conducted in Chile in 2016–2017 revealed a 3.0% seroprevalence for IgG antibodies against *C. burnetii*. Our findings suggest that Q fever was endemic to Chile before the documented outbreak of atypical pneumonia associated with *C. burnetii* infection reported in July 2017 in the three southern regions (La Araucanía, Los Ríos and Los Lagos) of the country [22]. Similar national seroprevalence levels were previously reported in the USA (3.1%) [14] and in The Netherlands (2.4%) [12], but lower than Australia (5.6%) [13]. Analysis of the characteristics of Chilean *C. burnetii* seropositive individuals revealed that seropositivity in the ≥65 years of age group was significantly higher than younger age groups. The reported linear increase in seropositivity to *C. burnetii* from the 24–44 year group onwards is consistent with the data from other national surveys in the Netherlands and USA surveys where similar linear increases in seropositivity with a peak in the older age groups [12,14]. The exception to this observation is the recently published Australian survey where *C. burnetii* seropositivity peaked in the middle years (50–59 years) before declining again in the older age groups [13]. Probably, the linear increase is due to accumulated exposure over the lifetime. Furthermore, in line with other nationwide surveys [12,13,14], *C. burnetii* seropositivity was higher in males than females in Chile. The sex gap in *C. burnetii* seroprevalence can be partly explained by increased occupational exposure to livestock by males compared to females [13].

Analysis of contextual risk factors associated with *C. burnetii* seroprevalence identified the southern macro-zone of Chile and residing in rural regions as significant risk factors for exposure. In Chile, these differences may be related to varying exposures by macro-zones, density population (urban/rural) or occupations lifestyle, economic development of the zone, but also attributable to other disease ecologic factors, such as the presence of vectors or reservoirs of the infection. Chile extends over 4300 km along the west side of South America and encompasses several climatic conditions, including the hyper-arid northern macro-zone, Mediterranean (central and metropolitan macro-zone) and hyper-humid regions (south macro-zone) [24]. This geographic diversity is home to many flora and fauna (ruminant or wild animals), which provides the basis for the country’s diversified agricultural, cattle raising and forestry industries. In this context, the three key regions of the southern macro-zone—the La Araucanía, Los Ríos and Los Lagos—have optimal geographical and climatic conditions for livestock and milk production at national levels [25]. Notably, *C. burnetii* was detected in bulk tank milk samples from dairy cattle in Chile (2.1% of the milk samples), indicating that the potential for transmission to humans exists [26]. The association between *C. burnetii* seropositivity and rural/urban regions is not new. A prospective cohort study of Q fever in older Australian adults found that living on a farm in an outer regional or remote area was a significant risk factor for *C. burnetii* exposure [27], reflecting the likely zoonotic origin of the infection and the occupational risk of exposure. Based on the identified risk factors and the documented *C. burnetii* seropositivity in this study, the southern regions of Chile must be considered as an important hot spot for Q fever risk in both humans and livestock.

The findings of this study need to be interpreted in light of some limitations. First, as only two years of ENS2016-2017 data were used, the small number of Q fever seropositive persons identified does not enable identifying more risk factors in certain subpopulations within Chile. Second, ENS2016-2017 is a nationally representative survey and is, therefore, not designed to give estimates in smaller geographic areas or specific high-risk individuals. In the near future, it is essential to complement the present findings with specific high-risk population seroprevalence studies, including subpopulations of individuals that are expected to have a higher occupational risk, such as farmers and other agricultural workers. Finally, variation in the kinetics of the immunoglobulins directed against *C. burnetii* antigens and the differences in diagnostic performance in available commercial Q fever immunoassay kits are well-known limiting factors in the planning of a *C. burnetii* seroprevalence study [28,29]. In this study, we attempted to minimize the challenges of *C. burnetii* diagnosis by before screening specimens collected as a part of the ENS survey, validating immunoassays against a serum panel composed of previously screened samples tested by the Australian Rickettsial Reference Laboratory (ARRL). This procedure provides confidence in the interpretation of immunoassay seropositivity reported in this study.

The presence of emerging or underdiagnosed zoonotic diseases, such as Q fever, is an international challenge. Fittingly, international collaboration was an essential component of this study, allowing the establishment of adequate epidemiological and diagnostic tools for determining accurate data on the seroprevalence of *C. burnetii* in the Chilean population. Our findings highlight the need for targeted risk-based Q fever surveillance in the areas identified at the highest risk of exposure by deploying follow-up seroprevalence surveys in the population most at-risk in these locations. Such seroprevalence studies within risk groups or risk areas in Chile could provide a useful alternative approach to complement routine surveillance data of *C. burnetii* across the country.

## 4. Materials and Methods

### 4.1. Population and Study Design

A cross-sectional serological study was performed using the Chilean National Health Survey 2016–2017 (ENS2016-2017) [23]. This cross-sectional survey is a nationwide tool, supported by the Departamento de Epidemiología, Ministerio de Salud de Chile, for public health epidemiological surveillance.

Briefly, 6233 persons were surveyed between August 2016 and March 2017 (Winter, Spring and Summer seasons), representing the general population (Chilean and foreign national) living in Chile. Chile’s national territory is located in the southern cone of South America. It has a total land size of 756,102 km^2^ and extends for more than 6435 km (17.8° S to 30 55° S) along the eastern edge of the Pacific Ocean. Chile is divided into 16 administrative regions with an average population density of 25.2 people/km^2^ per square km population density. However, this varies from region to region (474.85–0.99 range) [30]. The ENS2016-2017 survey, including women and men ≥ 15 years of age, was a stratified, multi-stage and clustered random sample of households at the national, regional and population density level (e.g., urban/rural). The absolute sampling error was 2.6% at the national level, with 95% CI and a relative error of less than 30% [23]. Trained interviewers and nurses applied health questionnaires, physical examinations and collected blood, serum and urine samples in successive household visits. All participants signed informed consent before participation in the ENS2016-2017 survey. For persons younger than 18 years, consent was obtained from the individual and parent or a legal representative.

### 4.2. Serological Screening and Confirmation of Phase I and II IgG antibodies against Coxiella burnetii

Sufficient sera from 5166 (82.9%) individuals were available from the ENS2016-2017 survey for *C. burnetii* serology. Samples were stored at the National Reference Laboratory, Public Health Institute of Chile at −80 °C until tested. Initial serological screening for *C. burnetii* IgG phase II antibodies was performed using a commercial Q fever enzyme-linked immunosorbent assay (ELISA), as per the manufacturer’s instructions (Q fever, IgG ELISA PanBio Inc., Columbia, MD, USA). Sera were diluted 1:100 with dilution buffer provided, and 100 µL of each diluted serum were added to 96-microwells coated with *C. burnetii* phase II antigen. Plates were processed with a fully automated ELISA processing system, according to the manufacturer’s instructions (Immunomat TM Fully automated SERION ELISA analyzer). The interpretation of ELISA assay results was performed following the protocol provided by the manufacturer. Briefly, the results were determined by comparison with a provided IgG reference sample, which contains a borderline level of IgG phase II antibody (cutoff calibrator). A positive serum was defined as having a sample absorbance/calibrator absorbance ratio of >1.1; an equivocal sample between ELISA ratio 0.9–1.1; and finally, a negative sample had a ratio of <0.9. All samples positive or equivocal by ELISA were then tested for the presence of IgG antibodies response to *C. burnetii* phase I and II antigens by a commercial indirect immunofluorescent antibody (IFA) assay, as per the manufacturer’s instructions (Focus Diagnostics, Inc., Cypress, CA, USA). Titration was carried out with dilutions according to a binary scale with an initial detection titer of 1/16. If reactive, a doubling dilution series (1/32 to 1/1024) of each serum was prepared. Sera positive and negative controls were included in each test. *C. burnetii* seropositivity was defined as a phase I or phase II IgG ≥ 1/32 by IFA. In a previous assessment of Q fever serological assays, we evaluated the sensitivity (0.8222, 95% CI 0.7471–0.8826) and specificity (0.8342, 95% CI 0.7751–0.8830) of the ELISA test for phase II IgG, using a panel, composed of a collection of Chilean serum samples previously tested by the ARRL [22].

### 4.3. Statistical Analyses

Estimates of variances for complex samples were calculated using the Taylor series linearization method. The results were expanded nationwide through expansion factors as the Fexp_EX1p_Corr, also used in the National Health Survey. The binomial logistic regression model was performed to analyze age, sex, macro-zone, and educational level on Q fever positivity. The significance level was set at 5%. All statistical analyses were conducted using the Statistical Package for the Social Sciences (SPSS, version 19) and the module for complex sample analyses.

## 5. Conclusions

The current study had defined the baseline serological prevalence of *C. burnetii* infection in Chile before the large Q fever outbreak detected in 2017 [22], including significantly higher levels of seropositivity in the southern macro-zones of this country. Following detecting the Chilean outbreak of Q fever in the latter regions, the Chilean Ministry of Health promoted and implemented public preventive measures to control this disease in livestock workers. Q fever is nevertheless likely to still pose an ongoing zoonotic disease threat in Chile, and it is critically important to maintain accurate national and regional surveillance. This knowledge will continue to facilitate developing efficient prevention strategies and implementing such prevention measures in a sustainable manner.

## Figures and Tables

**Table 1:** Reactivity of IgG antibodies against *Coxiella burnetii* in 5166 serum samples collected in the ENS 2016–2017 survey representing multiple regions of Chile.

Regions	Macro-Zones	Sample *n*	*C. burnetii* ELISA Positivity *n*	*C. burnetii* Confirmation IFA *n*	Congruence (%)	*C. burnetii* ELISA Equivocal *n*	*C. burnetii* Confirmation IFA *n*	Congruence (%)
Arica y Parinacota	North	251	7	5	71.4	2	2	100
Tarapacá		263	8	8	100	2	1	50.0
Antofagasta		246	7	4	57.1	3	3	100
Atacama		262	8	8	100	1	0	0.0
Coquimbo		279	13	12	92.3	4	1	25.0
Valparaíso	Central	539	17	17	100	9	5	55.6
L. Bdo. O’Higgins		282	9	9	100	4	3	75.0
Maule		309	10	7	70.0	3	0	0.0
Ñuble		119	3	3	100	0	0	0.0
Biobio		447	11	11	100	5	2	40.0
La Araucanía	South	266	11	11	100	6	5	83.4
Los Ríos		264	6	6	100	4	3	75.0
Los Lagos		278	10	10	100	4	1	25.0
Aysén		301	3	2	67	0	0	0.0
Magallanes		270	2	2	100	4	2	50.0
Metropolitan	Metropolitan	790	18	15	83	8	2	25.0
Total, *n*	-	5166	143	130	91	59	30	50.8

**Table 2:** Adjusted seroprevalence and corresponding 95% confidence intervals by characteristic data collected in the ENS2016-2017 survey.

Characteristics	Sample Size (*n*)	% Seroprevalence (95% CI)	*Expanded population* Seropositive Sample (*n*)
Overall	5164	3 (2.2–4.0)	409,480 (160)
Sex			
Female	3263	2.2 (1.4–3.5)	156,513 (86)
Male	1903	3.7 (2.5–5.4)	252,967 (74)
Age group, y			
15–24	674	2.4 (0.9–6.0)	63,668 (8)
24–44	1472	1.9 (1.0–3.8)	100,655 (26)
45–64	1720	3.4 (2.3–5.1)	143,631 (63)
65+	1254	5.6 (3.6–8.6)	101,525 (63)
Education level (Years completed)			
Low (<8)	1236	6.8 (3.9–11.4)	151,660 (63)
Middle (8–12)	2770	2.2 (1.6–3.2)	172,045 (72)
High (>12)	1114	2.2 (1.1–4.5)	172,045 (25)
Population density			
Urban	4344	2.6 (1.9–3.6)	317,968 (121)
Rural	820	5.9 (3.2–10.8)	91,512 (39)
Macro-zone			
North	1300	2.1 (1.4–3.2)	36,003 (44)
Central	1695	3.5 (2.2–5.4)	157,972 (57)
Metropolitan	790	1.8 (0.9–3.3)	100,357 (17)
South	1379	6.0 (3.3–10.6)	115,150 (42)

Adjusted for sex, age, an education level (low, middle and high), population density (urban and rural) and macro-zone (north, central, metropolitan and south).

## Data Availability

The data presented in this study are available on request from the corresponding author and the Chilean Ministry of Health. The data are not publicy due to privacy restriction.
